# Spatiotemporal Clustering Analysis and Risk Assessments of Human Cutaneous Anthrax in China, 2005–2012

**DOI:** 10.1371/journal.pone.0133736

**Published:** 2015-07-24

**Authors:** Wen-Yi Zhang, Li-Ya Wang, Xiu-Shan Zhang, Zhi-Hai Han, Wen-Biao Hu, Quan Qian, Ubydul Haque, Ricardo J. Soares Magalhaes, Shen-Long Li, Shi-Lu Tong, Cheng-Yi Li, Hai-Long Sun, Yan-Song Sun

**Affiliations:** 1 Institute of Disease Control and Prevention, Academy of Military Medical Science, Beijing, People’s Republic of China; 2 Navy General Hospital of PLA, Beijing, People’s Republic of China; 3 School of Public Health and Social Work, Queensland University of Technology, Brisbane, Australia; 4 Emerging Pathogens Institute, University of Florida, Gainesville, Florida, United States of America; 5 Department of Geography, University of Florida, Gainesville, Florida, United States of America; 6 School of Veterinary Science, The University of Queensland, Brisbane, Australia; 7 Children’s Health and Environment Program, Queensland Children’s Medical Research Institute, The University of Queensland, Brisbane, Australia; 8 Academy of Military Medical Science, Beijing, People’s Republic of China; University of Texas Medical Branch, UNITED STATES

## Abstract

**Objective:**

To investigate the epidemic characteristics of human cutaneous anthrax (CA) in China, detect the spatiotemporal clusters at the county level for preemptive public health interventions, and evaluate the differences in the epidemiological characteristics within and outside clusters.

**Methods:**

CA cases reported during 2005–2012 from the national surveillance system were evaluated at the county level using space-time scan statistic. Comparative analysis of the epidemic characteristics within and outside identified clusters was performed using using the χ^2^ test or Kruskal-Wallis test.

**Results:**

The group of 30–39 years had the highest incidence of CA, and the fatality rate increased with age, with persons ≥70 years showing a fatality rate of 4.04%. Seasonality analysis showed that most of CA cases occurred between May/June and September/October of each year. The primary spatiotemporal cluster contained 19 counties from June 2006 to May 2010, and it was mainly located straddling the borders of Sichuan, Gansu, and Qinghai provinces. In these high-risk areas, CA cases were predominantly found among younger, local, males, shepherds, who were living on agriculture and stockbreeding and characterized with high morbidity, low mortality and a shorter period from illness onset to diagnosis.

**Conclusion:**

CA was geographically and persistently clustered in the Southwestern China during 2005–2012, with notable differences in the epidemic characteristics within and outside spatiotemporal clusters; this demonstrates the necessity for CA interventions such as enhanced surveillance, health education, mandatory and standard decontamination or disinfection procedures to be geographically targeted to the areas identified in this study.

## Introduction

Anthrax is caused by the spore-forming *Bacillus anthracis* and is enzootic in many parts of Asia, sub-Saharan Africa, and some developing countries in the Mediterranean [1]. While cutaneous anthrax (CA) cases are rare in developed countries, recent outbreaks in the United States and European countries, and newly identified subcutaneous anthrax in injectable heroin users have heightened concerns about the disease [2–5]. Dormant endospores are resistant to drying, gamma radiation, and many disinfectants, and highly stable spores can stay preserved in some types of soil for decades [6,7]. Its environmental survival coupled with its ability to readily cause infection after inhalation, and the high mortality rate among resulting anthrax cases, *B*. *anthracis* is in the list of potential biological bioterrorism agents, as evidenced by the 2001 anthrax attacks in the USA [8–10].

Generally, human *B*. *anthracis* infections are caused by direct or indirect contacts with contaminated grazing herbivores or their products. In China, 97% of *B*. *anthracis* infections are the CA form [11]. Recently, CA outbreaks in some areas of Jiangsu and Liaoning, which were previously non-epidemic areas, have attracted the extensive attention from the general public and media. In addition, the future dynamics of CA transmission is a great public health concern. Therefore, it is essential to examine the spatial and temporal patterns of CA in China for the purpose of targeted surveillance and control.

A previous study identified significant clusters during an outbreak of anthrax in Saskatchewan by using a spatial statistic and geographic information system (GIS), which was conducive to identify the risk factors for the outbreak [12]. Moreover, a study in Georgia using passive surveillance data identified clusters of areas that were of historical importance for CA risk and new areas that represent (re)emergence [13]. This method has considerably advanced during the last two decades and has been widely applied to visualize epidemiological data, detect high-risk clusters, improve surveillance and establish efficient control measures for infectious diseases such as malaria [14], Japanese encephalitis [15], dengue fever [16], scrub typhus [17] and hemorrhagic fever with renal syndrome [18].

Fewer studies have explored the spatiotemporal patterns of CA and investigated the determinants of outbreaks [12,13]. While studies have been conducted in China investigating the CA epidemic characteristics at the province or municipality level, the spatiotemporal distribution of CA cases in the country is poorly understood [11,19].

This study aimed to investigate the nationwide epidemic characteristics of CA, detect clusters at the county level, and evaluate differences in the epidemic characteristics inside and outside identified clusters. The findings are important to plan the design and implementation of preemptive public health interventions for the local management of human CA cases.

## Materials and Methods

### Ethics Statement

This study was approved by the Ethics Committee of Beijing Institute of Disease Control and Prevention. In this study, all the data used were anonymous to protect patient’s confidentiality.

### Data Collection and Management

In China anthrax is a nationally reportable infectious disease. We analyzed disease surveillance data on human CA cases reported to the National Notifiable Disease Surveillance System during 2005–2012, including information on gender, age, occupation, place of residence, and date of illness onset and diagnosis. All human CA cases were diagnosed according to the unified criteria issued by the Ministry of Health of the People’s Republic of China [20]. The diagnostic criteria include a history of exposure and clinical manifestations (such as exposure of the skin of the head, neck or extremities resulting in erythema, papula, vesicles, and subsequently central necrosis, leaving a characteristic black eschar with a painless cutaneous lesion) and detection of a four-fold or greater increase in the level of *B*. *anthracis*-specific antibodies or isolation of *B*. *anthracis* from clinical specimens [21].

In our study, the 8–year data set of CA cases was integrated at the county level for spatiotemporal cluster analysis. To conduct a GIS-based analysis, the county-level polygonmap was obtained from the National Administration of Surveying, Mapping and Geoinformation. Demographic information, integrated in terms of the administrative county, was collected from the National Bureau of Statistics of China. All cases were geocoded and matched to the county-level layers of the polygon and paired with demographic information by administrative code using the ArcGIS software (version 9.3, ESRI, Redlands, CA).

### Spatiotemporal Cluster Analysis

We used Kulldorff’s space-time scan statistic to test whether the distribution of CA cases was random over space and time [22]. A discrete Poisson model was fitted to identify high-risk spatiotemporal clusters using the SaTScan software (version 9.1.1) [23]. SaTScan implements a series of variable-sized windows to detect the spatiotemporal clusters. The space-time scan statistic is defined by a cylindrical window with a circular (or elliptic) geographic base and with height corresponding to time [23].

In our study, circular scan windows were used to fit discrete Poisson models. The maximum spatial cluster size was set to 40% of the population at risk in the spatial window and a maximum temporal cluster size of 40% of the study period in the temporal window. Because anthrax was a seasonal disease, we made temporal adjustment for seasonality in our study. To adjust for the underlying distribution of the population, age and gender as the potential covariates from the population and cases files were included in our study. According to the criteria of National Bureau of Statistics of China, we divided the population and CA cases into 3 age groups: ≤ 15 years, 16–59 years, ≥ 60 years. The likelihood ratio tests were performed to test the significance of identified clusters and p-values were obtained through 999 Monte Carlo simulations [23]. Clusters with a p-value of < 0.05 were considered to be statistically significant. All the spatiotemporal clusters were mapped using ArcGIS version 9.3 (ESRI, Redlands, CA).

### Comparative Analysis of the Epidemiological Characteristics within and outside Clusters

To determine whether the epidemiologic characteristics of CA cases within and outside clusters were different, comparative analyses were performed between high-risk counties (spatiotemporal clusters detected by space-time scan statistic) and low-risk counties (not included in the spatiotemporal clusters) using the χ^2^ test or Kruskal-Wallis test. All statistical analyses were conducted using the SAS program (SAS Institute Inc., Cary, NC. Version 9.2)

## Results

### Descriptive Analysis

A total of 3,236 CA cases were reported from 298 counties of 19 provinces during January 2005–December 2012 in mainland China (a total of 2,922 counties). The majority of the cases (72.84%) were reported in male patients, and the incidence in males was much higher than that in females (χ^2^ = 601.24, p < 0.001). Furthermore, 41.10% and 45.46% of the cases were reported among shepherds and farmers, respectively. 2,046 (63.22%) cases were reported in persons aged 20–49 years. Grouping by age in 10–year intervals showed that 30–39 years group had the highest incidence (χ^2^ = 10.84, p < 0.001) and the fatality increased with age (Cochran-Armitage trend test: Z = 5.07, p < 0.001), with persons ≥ 70 years showing a fatality rate of 4.04%. A total of 35 CA-related deaths were reported during 2005–2012; farmers and shepherds accounted for 77.14% and 22.86% of these deaths, respectively.

**Fig 1:**
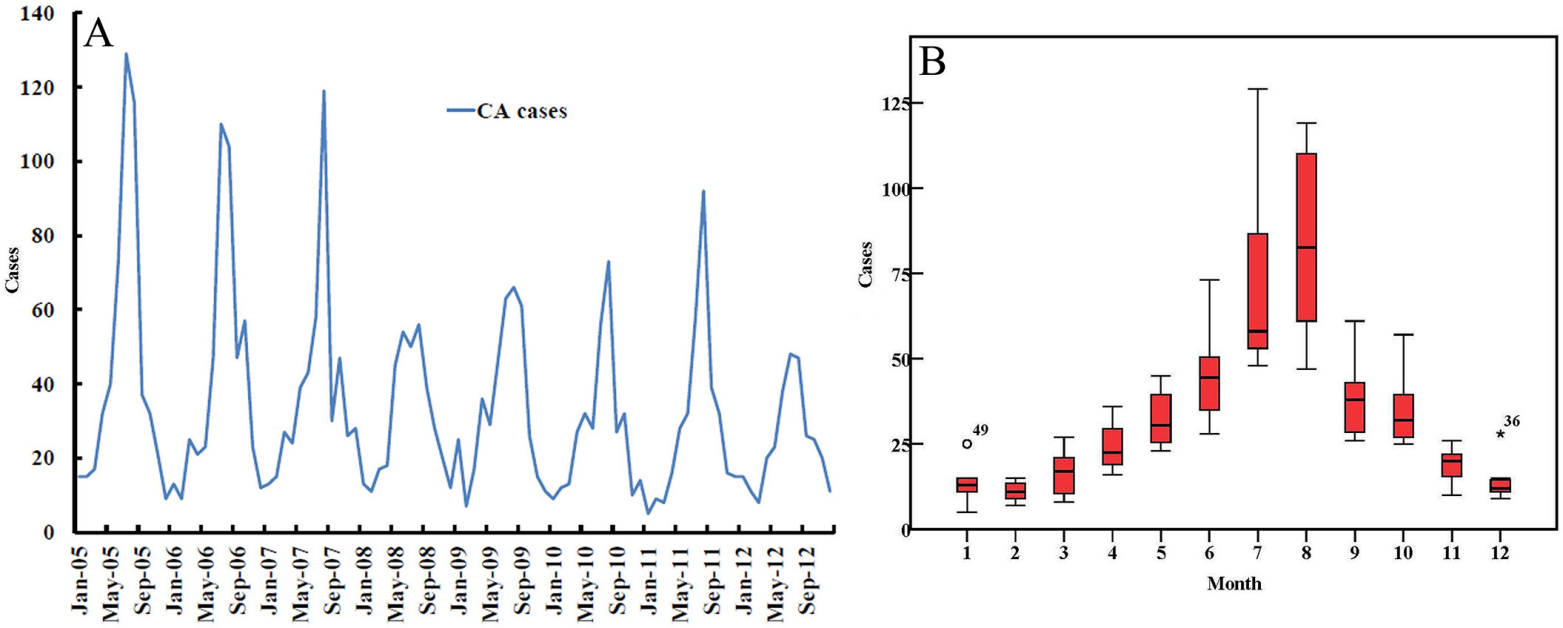
Epidemic curve, seasonal pattern and temporal clusters of human cutaneous anthrax in mainland China, 2005–2012. (A)The figure shows epidemic curve of monthly CA cases (blue line) and significant temporal clusters (red line) from 2005–2012; (B) The seasonal pattern of CA. The bottom and top of the box represents the lower quartile (P_25_) and the upper quartile (P_75_) respectively; the bottom, middle and top line is minimum, median and maximum value; dot and asterisk with number represent value of outlier and extreme outlier, respectively.

**Fig 2:**
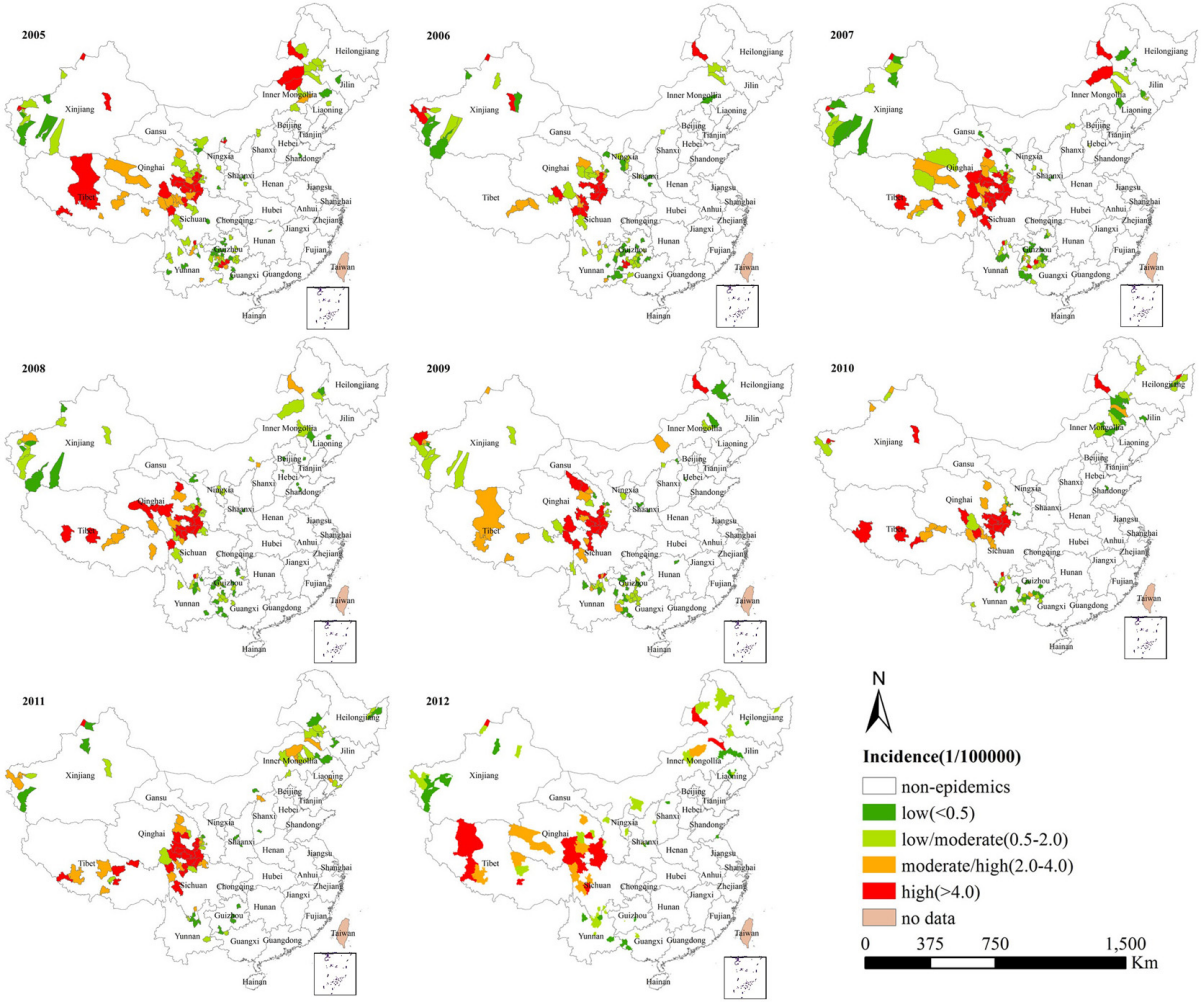
Annual incidence of human cutaneous anthrax in mainland China, 2005–2012.

Fig 1 depicts the temporal variation in monthly CA cases and the seasonal pattern of CA cases. CA cases occurred throughout the year, and the number of CA cases showed an initial increase from April followed by a peak in July–August and a decrease thereafter. The seasonal difference in CA occurrence was statistically significant (χ^2^ = 323.19, p < 0.001), with the highest occurrence noted in summer. Fig 2 shows yearly distributions of CA in mainland China from 2005 to 2012. Although there were different distributions of CA each year, the counties with the highest incidence were persistently located in the border areas of Sichuan, Gansu and Qinghai provinces (Fig 2).

### Spatiotemporal Cluster Analysis

**Fig 3:**
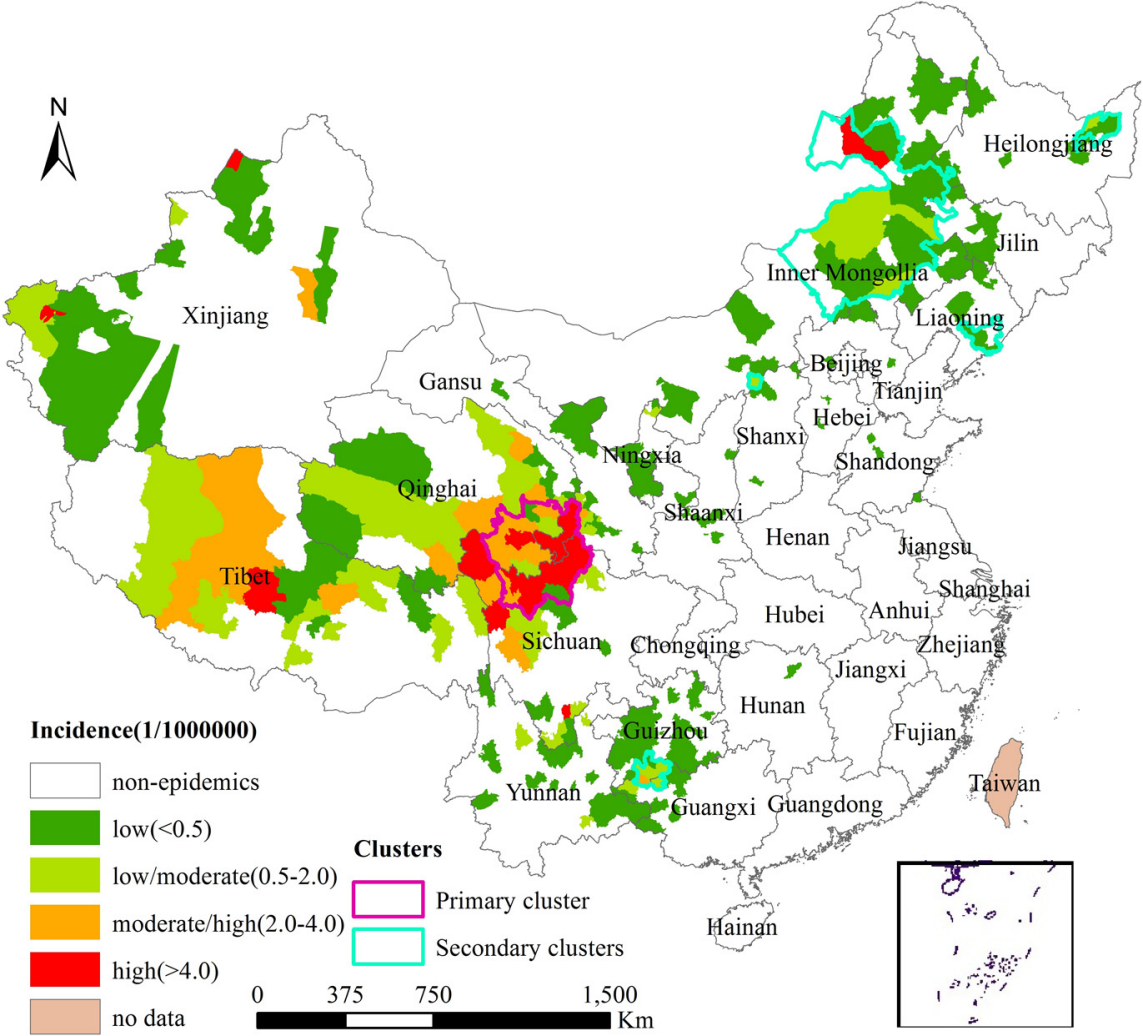
Spatiotemporal clusters of human cutaneous anthrax in mainland China, 2005–2012.

Space-time cluster analysis of CA from 2005 to 2012 detected a significantly high risk associated with spatiotemporal clusters. The primary cluster (cluster 1) contained 19 counties with a radius of 207 km from July 2006 to August 2009, mainly located at the border area of three provinces (Sichuan, Gansu, and Qinghai), including the top three counties with reported cases: Zoigee County, Maqu County, and Hongyuan County (Fig 3). Five significant secondary clusters were also identified with Relative Risk (RR) ranging from 21.94 to 1100.99 (Table 1).

**Table 1:** Space-time clusters of human cutaneous anthrax in China, 2005–2012.

Cluster	Radius(Km)	Time Frame	No. Counties	No. Obs	No. Exp	LLR	RR^#^
1	207	2006/07-2009/08	19	603	0.84	3421	879.27
2	49	2005/04-2007/11	6	195	1.51	760	137.18
3	378	2010/05-2012/10	24	76	3.54	161	21.94
4	91	2010/08	4	25	0.02	150	1100.99
5	64	2011/07-2011/08	4	33	0.13	149	250.62
6	0	2008/7-2011/8	1	9	0.11	30	80.79

No. Counties: number of counties in each cluster; No. Obs: number of observed cases; No. Exp: number of expected cases; LLR: Log Likelihood Ratios; RR: Relative Risk; Cluster 1: Primary cluster; Cluster 2–6: Secondary clusters

^#^P<0.05.

Spatiotemporal clusters usually had a high incidence per person-year; these clusters covered a very low proportion of the total population but a high proportion of the total CA cases during the clustering time (Table 2). Except for the Cluster 6, counties in these high-risk clusters covered less than 1% of the total population of China, but accounted for 8.61–40.91% of the total cases during the clustering time. For example, Cluster 1 had the incidence of 22.17/100,000 person-year, and accounted for 40.91% of the total cases during the clustering time, but covered only 0.07% of the population. Cluster 2 had the incidence of 4.39/100,000 person-year, and accounted for 13.72% of the total cases during the clustering time, but covered only 0.13% of the population.

**Table 2:** Incidence of human cutaneous anthrax, proportion of cases and population in spatiotemporal clusters in China, 2005–2012.

Cluster	Time Frame	Population	Incidence	% Cases	%Population
1	2006/07-2009/08	858160	22.17	40.91	0.07
2	2005/04-2007/11	1664107	4.39	13.72	0.13
3	2010/05-2012/10	5420981	0.56	8.61	0.42
4	2010/08	946889	33.00	34.25	0.07
5	2011/07-2011/08	2986631	6.50	22.00	0.23
6	2008/7-2011/8	128420	2.21	0.76	0.01

Incidence: cases per 100000 person year; %Population: the percentage of population living in cluster/the total population; %Cases: the percentage of cutaneous anthrax cases observed in cluster/the total cases during the same time.

### Comparative Epidemiology of Cases within and outside Spatiotemporal Clusters

Males accounted for the majority of cases in both high-risk and low-risk counties (Table 3). Compared with low-risk counties, cluster 1 had a higher percentage of females (χ^2^ = 8.21, p < 0.05), while cluster 2 and cluster 3 had higher percentages of males (χ^2^ = 17.03, p < 0.001; χ^2^ = 4.64, p < 0.05). With regard to the difference in median age in CA cases, it was lower in cluster 1 than in the low-risk counties (35 years vs. 38 years, Z = -2.80, p < 0.05), and it was higher in clusters 3–6 than in low-risk counties (p < 0.01).The majority of reported CA cases occurred in the local residents, while in cluster 4 there were 88% of the cases from the floating population. The concentration of local residents was more frequent in clusters 1 and 2 compared with low-risk counties (p < 0.01). Shepherds accounted for the majority of CA cases in cluster 1(85.07%); this percentage was significantly higher than that of each group (Fisher exact test, p < 0.001). The median number of days from illness onset to diagnosis was lower in cluster 1 than in low-risk counties (Z = -5.28, p < 0.001); further, it was higher in clusters 3–5 than in low-risk counties (p < 0.01).With regard to the distribution of the 35 fatalities, one death was from cluster 1; 9 deaths from cluster 2; 2 deaths from cluster 3; 23 deaths from low-risk counties, with a statistically significant difference (Fisher exact test, p < 0.001). The mortality rate in cluster 1 was lower than in low-risk counties (0.15% vs. 1.03%, Fisher exact test, p < 0.05), while the mortality rate in cluster 2 was higher than that in low-risk counties (4.62% vs. 1.03%, Fisher exact test, p < 0.001)(Table 3).

**Table 3:** Comparison of characteristics of cutaneous anthrax in high-risk counties and low-risk counties identified by Kulldorff’s space time cluster analysis in China, 2005–2012.

Variables		High-risk counties						Low-risk counties
		Cluster 1	Cluster 2	Cluster 3	Cluster 4	Cluster 5	Cluster 6	
Sex	Male,%	402 (66.67*)	168 (86.15^#^)	63 (82.89^#^)	22 (88.00)	28 (84.85)	8 (88.19)	1666 (72.59)
	Female,%	201 (33.33)	27 (13.85)	13 (17.11)	3 (12.00)	5 (15.15)	1 (11.11)	629 (27.41)
Age (Year)	Median (IR^✝^)	35* (25–46)	39 (28–50)	45^#^ (33–52)	43^#^ (38–51)	42^#^ (39–45)	56^#^(54–59)	38(27–49)
Occupation	Shepherd, %	513 (85.07^#^)	1 (0.51)	16 (21.05)	4 (16.00)	0 (0.00)	0 (0.00)	796 (34.68)
	Farmer, %	21 (3.48)	185 (94.87^#^)	55 (72.37^#^)	19 (76.00^#^)	28 (84.85^#^)	8 (88.19#)	1155 (50.33)
Address	Resident, %	594 (98.51^#^)	195 (100.00^#^)	69 (90.79)	3 (12.00)	32 (96.97)	8 (88.19)	2180 (94.99)
	Floating, %	9 (1.49)	0 (0.00)	7 (9.21)	22 (88.00^#^)	1 (3.03)	1 (11.11)	115 (5.01)
Days✝	Median (IR)	4.0* (2.0–5.4)	4.0 (3.0–7.0)	5.4^#^ (3.6–7.7)	5.4^#^ (5.3–6.6)	7.5^#^ (4.5–8.7)	5 (2.8–5.8)	4.2 (2.4–7.0)
Deaths	No. (Mortality, %)	1 (0.17*)	9 (4.62^#^ ^)^	2 (2.63)	0 (0)	0 (0)	0 (0)	23 (1)

* Significant difference with lower value in high-risk counties compared with low-risk counties (χ^2^ test, Fisher’s exact test or Z test with p<0.05)

# Significant difference with higher value in high-risk counties compared with low-risk counties (χ^2^ test or Z test with p<0.05)

^✝^ Interquartile range

✝ Days from illness onset to diagnosis.

## Discussion

The results of our study indicated that CA occurred throughout the year, with the lowest incidence reported in February and the highest incidence reported in August. There was significant seasonality between May/June and September/October. The seasonal variations in each year may be associated with exposure to hot weather, which may modify the resistance of the mucous membranes or skin of grazing animals to the pathogen, assist entry of the spores into the body of the animal, and reduce the host’s innate resistance to infection by increasing the infectivity of low doses of spores [24]. Furthermore, human would increase the chances of exposure to sick livestock or contaminated soil during these hot months. Such distinctive seasonal distribution has been reported in studies from other countries [25–28].

Our study identified six significant spatiotemporal clusters and the primary cluster persistently located in the border areas of Sichuan, Gansu, and Qinghai. These high-risk counties covered less than 1% of the total population of China during the clustering time. The identification of high-risk clusters indicated persistent infections of CA over space and time, which would be conducive to targeted control actions. It is believed that the high endemicity of CA in the high-risk areas identified in our study is closely associated with the work habits of farmers and shepherds and their contact with contaminated meat and soils [11,29]. Moreover, the space-time scan statistic identified one new cluster of CA in some parts of Liaoning province that were earlier non-epidemic. Similar patterns of outbreaks have been reported in Bangladesh and India [30,31].

Interestingly, the characteristics of CA cases in the high-risk and low-risk counties as identified by space-time cluster analysis were not similar. In cluster 1 where are typical pastoral areas, the majority of cases were younger, local, male individuals who were shepherds, living on agriculture and stockbreeding. Younger males were more likely to be involved in occupational exposure activities, such as skinning, butchering sick animals, and handling contaminated meat, whereas females are more likely to be involved in house-work and agriculture activities or cooking contaminated meat. Therefore, the occurrence of CA among females may be associated with exposure to contaminated soil, which is a continuous source of infection [19]. Another interesting finding is that no cases of gastrointestinal anthrax were reported, even though people living in these areas have the custom of eating sick or dead livestock because they are unwilling to discard them, and similar findings were reported in a study from Turkey [26]. Poverty, insufficient livestock vaccination coverage, lack of awareness about CA transmission from animals to humans, and behavioral and cultural factors all contribute to these outbreaks and epidemics in China [26,32–34].

In our study we found high morbidity, low mortality and quick confirmation from illness onset to diagnosis in the natural foci especially in cluster 1, which may be related with better awareness among residents and physicians about clinical manifestations because of a long history of epidemics. Higher morbidity and mortality were noted in the border areas of Guangxi and Guizhou provinces (cluster 2) between April 2005 and November 2007. This trend has been reported in some parts of Turkey too [26], and may be associated with the implementation of effective control measures. However, the real cause should be further studied, as it would be useful for disease control in other regions. Older, male farmers were predominately found in clusters 3–6 and they had a longer intervals from illness onset to diagnosis; this may be associated with changes in sociocultural practices. In these atypical areas, the lack of awareness about the disease may be the reason for a longer period of time from illness onset to diagnosis.

Our analyses identified clusters of counties that are historically known as natural foci of CA as well as new counties that represent areas of (re)emergence; furthermore, different epidemic characteristics of CA were detected inside and outside spatiotemporal clusters. These findings can be useful to formulate public health interventions aimed at target prevention and control of CA and increasing awareness about the disease. Given the limited resources available, different targeted efforts in different areas are needed. Prevention and control measures including scientific self-protection, supervision, vaccination campaigns among domestic animals, decontamination of soil and environmental management should be prioritized in natural foci areas to increase the effectiveness of such programs. Health education and promotion campaigns among both residents and physicians are a priority in atypical areas. It is important to strengthen cooperation among related health administration, such as intercommunication between the authorities in charge of livestock cases and human cases, in order to quickly respond to epidemics or outbreaks.

There are some limitations in this study. First, the circular scan window in the space-time scan statistic may have detected larger clusters containing low-risk counties. This is unavoidable, especially in border or irregular areas, because of the algorithm used [22]. Therefore, the incidence in cluster 3 was lower than that in other spatiotemporal clusters. In fact, cluster 3 contained true and false high-risk counties. Second, data in this study are obtained from a passive surveillance system, which could indicate that the data are underreported. Third, we created the risk maps based on reports of human anthrax cases, which is useful from the viewpoint of national public health surveillance and prevention strategies, but it is not noteworthy outside of this context. Further studies are necessary to quantify the role of known environmental, such as soil factors (pH, calcium ion concentrations, soil type, hydrological data, and mean surface temperature) and socio-environmental factors (temperature, relative humidity, rainfall, land cover, working place, exposure, custom, and control measures) in the spatial variation identified in this study [7,35–38].

## Conclusion

This study investigated the heterogeneity of CA occurrence over space and time in China and described the different epidemiological characteristics in high-risk and low-risk counties during 2005–2012. Based on this information, informed control and prevention measures focused on high-risk areas could provide more effective interventions. Given the limited resources available, different areas should have different priorities with regard to preferable surveillance, health education, mandatory and standard decontamination and disinfection procedures, in order to help reduce the occurrence of CA in China.

## References

[1] Doganay M, Metan G, Alp E. A review of cutaneous anthrax and its outcome. J Infect Public Health. 2010;3(3):98–105. 10.1016/j.jiph.2010.07.004 20869669

[2] Ringertz SH, Hoiby EA, Jensenius M, Maehlen J, Caugant DA, Myklebust A, et al Injectional anthrax in a heroin skin-popper. Lancet. 2000;356(9241):1574–5. 11075776 10.1016/s0140-6736(00)03133-0

[3] Ramsay CN, Stirling A, Smith J, Hawkins G, Brooks T, Hood J, et al An outbreak of infection with Bacillus anthracis in injecting drug users in Scotland. Euro surveillance. 2010;15(2). 10.2807/ese.15.02.19465-en 20085694

[4] Holzmann T, Frangoulidis D, Simon M, Noll P, Schmoldt S, Hanczaruk M, et al Fatal anthrax infection in a heroin user from southern Germany, June 2012. Euro surveillance. 2012;17(26). 22790532

[5] Palmateer NE, Ramsay CN, Browning L, Goldberg DJ, Hutchinson SJ. Anthrax infection among heroin users in Scotland during 2009–2010: a case-control study by linkage to a national drug treatment database. Clin infect dis. 2012; 55(5): 706–10. 10.1093/cid/cis511 22618565

[6] Inglesby TV, Henderson DA, Bartlett JG, Ascher MS, Eitzen E, Friedlander AM, et al Anthrax as a biological weapon: medical and public health management. Working Group on Civilian Biodefense. JAMA. 1999;281(18):1735–45. 10328075 10.1001/jama.281.18.1735

[7] World Health Organization. Anthrax in humans and animals—fourth edition. Available: http://www.who.int/csr/resources/publications/AnthraxGuidelines2008/en/. Accessed: 2008. 26269867

[8] Jernigan JA, Stephens DS, Ashford DA, Omenaca C, Topiel MS, Galbraith M, et al Bioterrorism-related inhalational anthrax: the first 10 cases reported in the United States. Emerg infect dis. 2001;7(6):933–44. 11747719 10.3201/eid0706.010604 PMC2631903

[9] Borio L, Frank D, Mani V, Chiriboga C, Pollanen M, Ripple M, et al Death due to bioterrorism-related inhalational anthrax: report of 2 patients. JAMA. 2001; 286(20):2554–9. 11722269 10.1001/jama.286.20.2554

[10] Kyriacou DN, Stein AC, Yarnold PR, Courtney DM, Nelson RR, Noskin GA, et al Clinical predictors of bioterrorism-related inhalational anthrax. Lancet. 2004; 364(9432):449–52. 15288744 10.1016/S0140-6736(04)16769-X

[11] Wang LM, Li F, Zhu XP. Epidemic of anthrax in Sichuan Province, 2004–2006. J Prev Med Inf. 2011;27(10):792–6.

[12] Epp T, Argue C, Waldner C, Berke O. Spatial analysis of an anthrax outbreak in Saskatchewan, 2006. Can Vet J. 2010;51(7):743–8. 20885827 PMC2885116

[13] Kracalik I, Malania L, Tsertsvadze N, Manvelyan J, Bakanidze L, Imnadze P, et al Human cutaneous anthrax, Georgia 2010–2012. Emerg infect dis. 2014; 20(2): 261–4. 10.3201/eid2002.130522 24447721 PMC3901487

[14] Zhang W, Wang L, Fang L, Ma J, Xu Y, Jiang J, et al Spatial analysis of malaria in Anhui province, China. Malaria j. 2008;7:206. 10.1186/1475-2875-7-206 PMC2572066 18847489

[15] Wang LY, Zhang WY, Ding F, Hu WB, Soares Magalhaes RJ, Sun HL, et al Spatiotemporal patterns of Japanese encephalitis in China, 2002–2010. PLoS negl trop dis. 2013;7(6):e2285 10.1371/journal.pntd.0002285 23819000 PMC3688550

[16] Li Z, Yin W, Clements A, Williams G, Lai S, Zhou H, et al Spatiotemporal analysis of indigenous and imported dengue fever cases in Guangdong province, China. BMC infect dis. 2012;12:132 10.1186/1471-2334-12-132 22691405 PMC3412724

[17] Zhang WY, Wang LY, Ding F, Hu WB, Soares Magalhaes RJ, Sun HL, et al Scrub typhus in mainland China, 2006–2012: the need for targeted public health interventions. PLoS negl trop dis. 2013;7(12):e2493 10.1371/journal.pntd.0002493 24386495 PMC3873277

[18] Fang LQ, Zhao WJ, de Vlas SJ, Zhang WY, Liang S, Looma CW, et al Spatiotemporal dynamics of hemorrhagic fever with renal syndrome, Beijing, People's Republic of China. Emerg infect dis. 2009;15(12):2043–5. 10.3201/eid1512.081078 19961697 PMC3044508

[19] Huang DH, Liang JM, Lu CF, Qin SY. Investigation into the prevalent status and factors associated with anthrax in 2002–2003 in Guangxi. China Trop Med. 2005; 5(3):456–7.

[20] China CDC. National anthrax monitoring program. Available: http://www.chinacdc.cn. Accessed: 2005.

[21] Swartz MN. Recognition and management of anthrax—an update. N Engl J Med. 2001;345(22):1621–6. 11704686 10.1056/NEJMra012892

[22] Kulldorff M. A spatial scan statistic. Commun Stat Theory Methods. 1997;26:1481–96.

[23] Kulldorff M. SaTScan User Guide for version 9.0. Available: http://www.satscan.org. Accessed: 2010.

[24] Clegg SB, Turnbull PC, Foggin CM, Lindeque PM. Massive outbreak of anthrax in wildlife in the Malilangwe Wildlife Reserve, Zimbabwe. Vet Rec. 2007;160(4):113–8. 17259452 10.1136/vr.160.4.113

[25] Demirdag K, Ozden M, Saral Y, Kalkan A, Kilic SS, Ozdarendeli A. Cutaneous anthrax in adults: a review of 25 cases in the eastern Anatolian region of Turkey. Infection. 2003;31(5):327–30. 14556058 10.1007/s15010-003-3169-3

[26] Ozkurt Z, Parlak M, Tastan R, Dinler U, Saglam YS, Ozyurek SF. Anthrax in eastern Turkey, 1992–2004. Emerg infect dis. 2005;11(12):1939–41. 16485484 10.3201/eid1112.050779 PMC3367647

[27] Ashkenazi-Hoffnung L, Kaufman Z, Bromberg M, Block C, Keller N, Dictar R, et al Seasonality of Bacillus species isolated from blood cultures and its potential implications. Am J Infect Control. 2009;37(6):495–9. 10.1016/j.ajic.2008.08.008 19162377

[28] Chikerema SM, Pfukenyi DM, Matope G, Bhebhe E. Temporal and spatial distribution of cattle anthrax outbreaks in Zimbabwe between 1967 and 2006. Trop Anim Health Prod. 2012;44(1):63–70. 10.1007/s11250-011-9888-z 21701924

[29] Chirundu D, Chihanga S, Chimusoro A, Chirenda J, Apollo T, Tshimanga M. Behavioural factors associated with cutaneous anthrax in Musadzi area of Gokwe North, Zimbabwe. Cent Afr J Med. 2009;55(9–12):50–4. 21977844 10.4314/cajm.v55i9-12.63640

[30] Siddiqui MA, Khan MA, Ahmed SS, Anwar KS, Akhtaruzzaman SM, Salam MA. Recent outbreak of cutaneous anthrax in Bangladesh: clinico-demographic profile and treatment outcome of cases attended at Rajshahi Medical College Hospital. BMC research notes. 2012;5:464 10.1186/1756-0500-5-464 22929128 PMC3493280

[31] Ray TK, Hutin YJ, Murhekar MV. Cutaneous anthrax, West Bengal, India, 2007. Emerg infect dis. 2009;15(3):497–9. 10.3201/eid1503.080972 19239777 PMC2666291

[32] Mwenye KS, Siziya S, Peterson D. Factors associated with human anthrax outbreak in the Chikupo and Ngandu villages of Murewa district in Mashonaland East Province, Zimbabwe. Cent Afr J Med. 1996;42(11):312–5. 9130412

[33] Zhang TL, Cui LL, Li L, Zhang ML, Qi F, Ying L, et al Investigation of an outbreak of cutaneous anthrax in Banlu village, Lianyungang, China, 2012. Western Pac Surveill Response J. 2012;3(4):12–5. 10.5365/WPSAR.2012.3.4.005 23908932 PMC3729087

[34] Kracalik IT, Malania L, Tsertsvadze N, Manvelyan J, Bakanidze L, Imnadze P, et al Evidence of local persistence of human anthrax in the country of georgia associated with environmental and anthropogenic factors. PLoS negl trop dis. 2013;7(9):e2388 10.1371/journal.pntd.0002388 24040426 PMC3764226

[35] Woods CW, Ospanov K, Myrzabekov A, Favorov M, Plikaytis B, Ashford DA. Risk factors for human anthrax among contacts of anthrax-infected livestock in Kazakhstan. Am J Trop Med Hyg. 2004;71(1):48–52. 15238688

[36] Blackburn JK, McNyset KM, Curtis A, Hugh-Jones ME. Modeling the geographic distribution of Bacillus anthracis, the causative agent of anthrax disease, for the contiguous United States using predictive ecologicalniche modeling. Am J Trop Med Hyg. 2007;77(6):1103–10. 18165531

[37] Schuch R, Fischetti VA. The secret life of the anthrax agent Bacillus anthracis: bacteriophage-mediated ecological adaptations. PloS one. 2009;4(8):e6532 10.1371/journal.pone.0006532 19672290 PMC2716549

[38] Joyner TA, Lukhnova L, Pazilov Y, Temiralyeva G, Hugh-Jones ME, Aikimbayev A, et al Modeling the potential distribution of Bacillus anthracis under multiple climate change scenarios for Kazakhstan. PloS one. 2010;5(3):e9596 10.1371/journal.pone.0009596 20231894 PMC2834750

